# Protein disulfide isomerase A3 as novel biomarker for endometrial cancer

**DOI:** 10.3389/fonc.2023.1247446

**Published:** 2023-10-16

**Authors:** Fanrong Yu, Xin Liu, Min Li, Xiufen Liu, Xintai Wang, Meixiang Guo

**Affiliations:** ^1^ Department of Obstetrics and Gynecology, Shanghai Jiao Tong University Affiliated Sixth People's Hospital South Campus, Shanghai, China; ^2^ Department of Pathology, Shanghai Jiao Tong University Affiliated Sixth People's Hospital South Campus, Shanghai, China; ^3^ School of Information Science and Technology, Dalian Maritime University, Dalian, China; ^4^ 2D Material Lab, Zhejiang Mashang Technology Research Institute, Cangnan, Wenzhou, Zhejiang, China; ^5^ Department of General Practice, Shanghai Jiao Tong University Affiliated Sixth People's Hospital South Campus, Shanghai, China

**Keywords:** endometrial cancer, novel biomarker, prognosis, PDIA3, protein expression

## Abstract

**Objective:**

This study aims to investigate the potential of PDIA3 as a novel prognostic biomarker and therapeutic target for Endometrial Cancer (EC) with the ultimate goal of improving survival rates in EC patients.

**Methods:**

This study employed a combination of public database analysis and clinical tissue sample assays. The analysis included comparing the gene expression of PDIA3 between EC and adjacent paracancerous tissues, investigating this expression status using qPCR and immunohistochemistry (IHC) assays, studying the correlation of expression with different parameters using Chi-square test, Cox Regression, and log-rank test, as well as exploring the PDIA3-related immune infiltration and metabolic pathway using TIMER and GSEA.

**Results:**

The analysis of public datasets revealed that PDIA3 mRNA and protein expression was significantly higher in EC tissues compared to adjacent tissues (P = 4.1e-03, P = 1.95e-14, and P = 1.6e-27, respectively). The qPCR analysis supported this finding (P = 0.029). IHC analysis revealed a significant increase in PDIA3 expression in endometrial cancer (EC) tissues compared to adjacent normal tissues (P = 0.01). Furthermore, PDIA3 expression showed significant correlations with cancer stage and tumor grade. Multivariate Cox regression analysis suggested that the PDIA3 gene holds promise as a prognostic factor for EC patients (HR = 0.47, 95% CI [0.27, 0.82], P = 0.008). The results from TIMER demonstrated a positive correlation between PDIA3 and tumor-infiltrating CD8 T cells and macrophages, and a negative correlation with tumor-infiltrating CD4 T cells. Additionally, the GSEA results indicated that PDIA3 overexpression was associated with various metabolic processes in EC patients.

**Conclusion:**

PDIA3 has been validated as a potential biomarker for EC, and its expression is further associated with pathological staging and prognosis.

## 1 Introduction

Endometrial cancer, which originates from the endometrial epithelium, is the sixth most common cancer among women worldwide and is associated with increasing mortality rates. In 2020 alone, there were over 417,000 new cases reported, resulting in 97,000 deaths (1, 2). Consequently, this cancer poses a significant threat to women’s health, particularly in developing countries (3, 4). While postoperative radiotherapy and chemotherapy are commonly used treatments for endometrial cancer patients (5), their efficacy varies among individuals, and the toxicity associated with these therapies cannot be ignored (6, 7). Therefore, there is an urgent need to develop new treatment targets for endometrial cancer to improve patient outcomes.

One potential target is Protein disulfide isomerase A3 (PDIA3), also known as GRP58, ERp57, or ERp60, which is an important component of major histocompatibility complex type (MHC) 1 (8, 9). PDIA3 belongs to the protein disulfide isomerase (PDI) gene family and primarily participates in calreticulin activity (10, 11). Recent studies have shown that PDIA3 is upregulated in various cancers, including breast, prostate, ovarian, glial cell carcinoma, and cervical adenocarcinoma, and is involved in their development, progression, and response to chemotherapy (12, 13). However, the role of PDIA3 in endometrial cancer remains unknown. In this study, we aimed to assess the clinical significance of PDIA3 in endometrial cancer by evaluating its expression in public datasets and primary tumor samples from participating hospitals, with the goal of identifying its potential as a novel biomarker.

Our research involved introducing PDIA3 as a novel biomarker for endometrial cancer and validating its expression using public datasets and primary tumor tissues. Furthermore, we examined the association between PDIA3 protein expression and clinical-pathological parameters of endometrial cancer. Finally, we evaluated the prognostic value of PDIA3 in endometrial cancer.

## 2 Materials and methods

### 2.1 Acquisition of public datasets

The GSE17025 dataset, which includes 12 normal samples and 91 EC tissues, was downloaded from the GEO database (https://www.ncbi.nlm.nih.gov/gds). RNA sequencing and corresponding clinical data were downloaded from The Cancer Genome Atlas (TCGA) database (https://portal.gdc.cancer.gov/). The corresponding clinical data included age, clinical stage, histological grade, histological type, number of pregnancies, menopause status, diabetes, hypertension, neoadjuvant treatment, radiation therapy, and living status. PDIA3 protein level expression data were collected from the CPTAC database (https://cptac-data-portal.georgetown.edu/). The survival information from the GEO dataset was obtained from the Kaplan-Meier website (https://kmplot.com/analysis/).

### 2.2 RT-qPCR of clinical tissue specimens after surgery

Ten tissue specimens (five tumors and five normal) were obtained from 5 patients with EC, who underwent surgical treatment at the Shanghai Jiao Tong University Affiliated Sixth People’s Hospital South Campus from January 2020 to January 2021. These 5 patients underwent surgical treatment after diagnostic curettage and none of them had received radiotherapy, chemotherapy, or hormone therapy before surgery. Pathological examination after surgery confirmed that they were EC. The specimens were rinsed with PBS and snap-frozen in liquid nitrogen and stored until use. The samples used were approved by the Ethics Committee of the South Campus Affiliated Sixth People’s Hospital of Shanghai Jiao Tong University.

Total RNA was extracted from EC tissues and adjacent tissues using TRIzol^®^ reagent, according to the manufacturer’s instructions. The tissues were frozen in liquid nitrogen and homogenized. The integrity of large RNA was confirmed by 1% denatured agarose gel electrophoresis (Sigma Aldrich) electrophoresis. RT-qPCR was performed to determine PDIA3 expression levels. According to the manufacturer’s instructions, RNA was reverse-transcribed to cDNA using an mRNA reverse transcription kit. The cDNA (10 ng) was then used as a template to amplify mature PDIA3. RT-qPCR was carried out using an ABI 7500 thermocycler (ABI 7500; Applied Biosystems Life Technologies, Foster City, CA, USA), according to the manufacturer’s instructions. The primers used were from Shanghai Sheng Gong Co., Ltd. (Shanghai, China), as follows: PDIA3, forward 5′-GCCTCCGACGTGCTAGAAC-3′ and reverse 5′-GCGAAGAACTCGACGAGCAT-3′; GAPDH, forward 5′-GTCAAGGCTGAGAACGGGAA-3′ and reverse 5′-AAATGAGCCCCAGCCTTCTC-3′. To determine the expression levels of PDIA3 mRNA, an SYBR Ex Taq kit was used for PCR amplification with GAPDH as an endogenous control gene. The PCR cycling conditions were as follows: 94°C for 3 min, followed by 40 cycles at 94°C for 30 s, 60°C for 30 s, and 72°C for 30 s. Relative expression levels were analyzed using the 2^ΔΔCt^ method (14).

### 2.3 Immunohistochemistry staining data from tissue microarray and primary samples

The PDIA3 protein was evaluated by tissue microarray (TMA). An EC TMA (HUteA060CS01 and HUteA045PG01) was purchased from Shanghai Outdo Biotechnology Company. The TMA contained 49 EC tissues and 37 adjacent non-tumor endometrial tissues.

The expression of the PDIA3 protein in tissue was evaluated by immunohistochemistry (IHC) using a mouse monoclonal PDIA3 antibody (5% BSA PBS dilution, 1:200, Abcam Biotechnology, UK). Paraffin-embedded TMA sections were heated to 60°C using a thermostat and then incubated for 20 min. After baking, tissue slices were placed in xylene and dewaxed twice for 5 min. Sections were rehydrated through graded concentrations of ethanol, heated in 10 mmol/L pH 6.0 citrate buffer in a steamer for 30 min, and then cooled to 25°C or 30 min for antigen retrieval. Next, 1.5% horse serum was used to block the sections for 30 min, after which 50 μL of primary antibody (diluted 1:200) was added to each section and then incubated overnight at 4°C. A 50-μL volume of secondary antibody was added and each section was incubated for 60 min at room temperature. Freshly prepared DAB working solution (50 μL) was then added to each slice and the color was allowed to develop for 3 to 5 min. Each slice was counterstained with hematoxylin, dehydrated, and fixed. Staining intensity scores were examined and scored by two pathologists with more than 15 years of experience as follows: The cells are scored on a 4-point scale based on staining intensity. No positive staining (negative) is scored as 0, pale yellow (weak positive) is scored as 1, brown-yellow (positive) is scored as 2, and brownish-brown (strong positive) is scored as 3. The percentage of positive cells is scored on a 4-point scale. ≤25% is scored as 1, 26%-50% is scored as 2, 51%-75% is scored as 3, and >75% is scored as 4.The final score is calculated by multiplying the scores from both criteria (15). We conducted quantitative analysis of the expression differences of PDIA3 protein using Image Pro Plus 6.0 software. The mean density value was calculated by measuring the integrated optical density (IOD) and area values of each image, according to the formula: mean density = IOD/area.

### 2.4 Evaluation of tumor-infiltration immune cells by TIMER

TIMER was used to analyze immune infiltrates in a spectrum of cancer types (https://cistrome.shinyapps.io/timer/) (16). TIMER uses a statistical method termed deconvolution, to approximate the number of tumor-infiltrating immune cells. We analyzed the expression of PDIA3 in endometrial carcinoma and its correlation with immune-infiltrating cells, including CD4+ T cells, CD8+ T cells, B cells, dendritic cells, macrophages, and neutrophils. We evaluated the correlation between immune infiltrates and gene expression, mutation status, somatic CNV, and clinical outcome.

### 2.5 Single gene GSEA

Gene set enrichment analysis (GSEA) version 4.0.3. (http://www.broadinstitute.org/gsea) was used to explore pathways related to PDIA3 expression levels in EC. If most of a gene set exhibited high expression accompanied by a high-risk score, this gene set had a positive enrichment score and was defined as “enriched”. In addition, c2.cp.kegg.v7.1.symbols.gmt. Human_ENSEMBL_Gene_ID_MSigDB.v7.0. The chip and the normalized enrichment score (NES) were calculated. The GSEA program was run with 1000 permutations to estimate statistical significance. A p-value < 0.01 was selected to indicate statistically significant pathways associated with PDIA3 expression.

### 2.6 Statistical analysis

Statistical analysis and visualization were performed using R software (version 4.3.3) and SPSS software (version 26, IBM Corp., Armonk, NY, USA) to analyze the data. Chi-square tests were used to evaluate the association between PDIA3 expression and clinicopathological parameters. Univariate and multivariate Cox regression analyses were performed to examine the independent prognostic value of PDIA3 expression in terms of overall survival (OS). The low and high expression groups of PDIA3 were classified based on a median expression. The survival distribution was estimated using Kaplan–Meier analysis and the log‐rank test was applied to evaluate the differences between the stratified groups. Statistical significance was defined as a two-tailed P value < 0.05.

## 3 Results

### 3.1 Workflow and study samples

**Figure 1:**
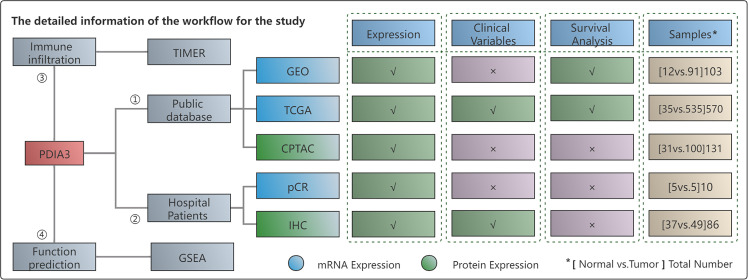
The workflow of this study. "*" represents the total number of (Normal vs Tumor) cases. In other words, it indicates that the number listed below includes both normal and tumor cases. "✓" represents the content that is present in the text. "x" represents the absence of content in the text.

**Figure 1** provides a flow chart outlining the study design, and it includes information on the enrolled patients. We analyzed the GSE17025 dataset from the GEO database, which included 12 normal samples and 91 EC tissues. Additionally, we utilized the TCGA database, which provided a total of 570 patient samples consisting of 35 normal tissues and 535 cancer tissues. To verify our results, we downloaded PDIA3 protein expression data from the CPTAC database, which included 131 patients. Furthermore, we obtained 5 matched EC and paracancerous tissues from our hospital, which were used for RT-qPCR analysis. For IHC, we used a total of 86 samples, including 37 normal tissues and 49 tumor tissues.

### 3.2 Upregulation of PDIA3 in endometrial cancer

**Figure 2:**
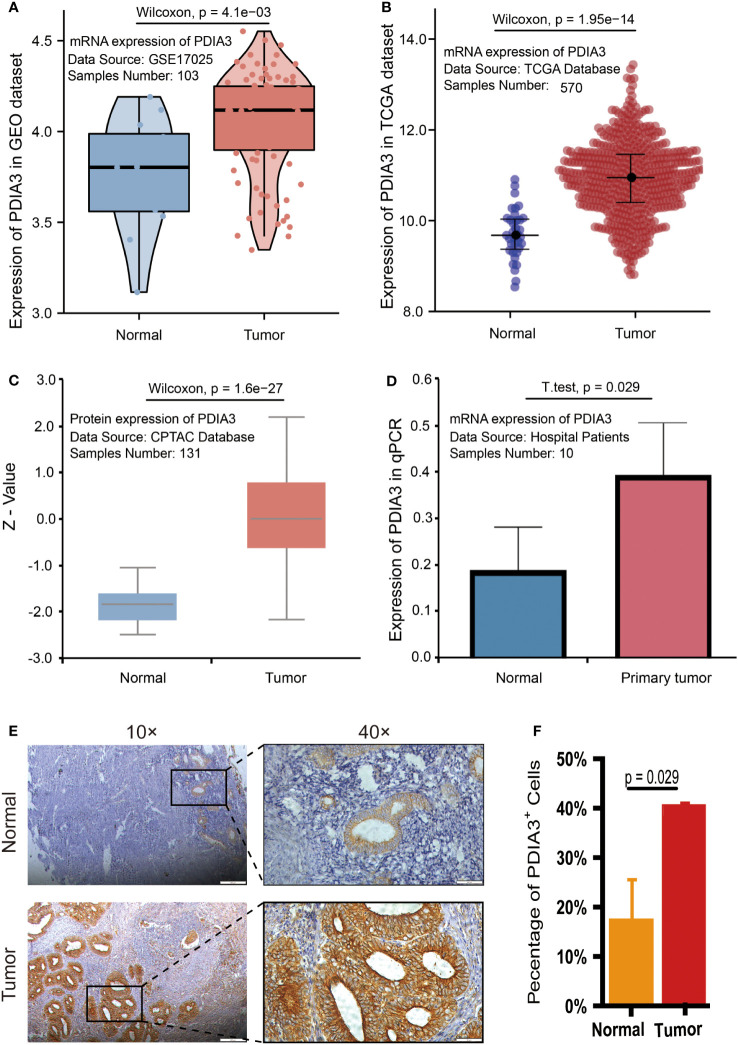
PDIA3 expression in public database: **(A)** PDIA3 expression in GSE17025 dataset, **(B)** PDIA3 expression in TCGA database, **(C)** PDIA3 expression in CPTAC database. PDIA3 expression in clinical samples of EC: **(D)** PDIA3 expression in 5 matched adjacent and EC tissues; **(E)** PDIA3 protein was localized to the cell membrane and minimally in certain parts of the cytoplasm. Positive staining with a staining score of 3+ in tumor tissue. Weak positive staining with a staining score of + in normal tissue, **(F)** The percentage of PDIA3 positive expression in EC and normal tissue.

We present the evidence for upregulation of PDIA3 in endometrial cancer in **Figure 2**. Based on the expression results obtained from the GSE17025 dataset, it was determined that PDIA3 expression in endometrial cancer (EC) tissues was significantly higher compared to adjacent samples (P = 4.1E-03) (**Figure 2A**). This finding was consistent with the results obtained from the TCGA-UCEC dataset (P = 1.95E-14) (**Figure 2B**). Additionally, the upregulation of PDIA3 protein expression in primary tumor tissues compared to normal tissues was verified using the CPTAC protein database (P = 1.6E-27) (**Figure 2C**). To investigate the expression of PDIA3 protein in clinical samples of endometrial cancer, RT-qPCR analysis was conducted on five matched EC and paracancerous tissues obtained from our hospital. The results confirmed a higher expression of PDIA3 in EC tissues compared to normal ones (P = 0.029) (**Figure 2D**). A tissue microarray comprising 49 endometrial cancer tissues and 37 adjacent non-cancerous tissues was employed for immunohistochemical (IHC) staining. The immunostaining results in **Figure 2E** indicated that the PDIA3 protein exhibited a predominantly membranous localization, with minimal cytoplasmic staining observed in certain regions. Comparative analysis revealed a significantly higher expression of PDIA3 in the endometrial cancer (EC) tissue compared to the corresponding normal tissue (P = 0.029), underscoring a statistically significant difference between the two groups as depicted in **Figure 2F**. The percentage of PDIA3-positive expression in the EC group and the adjacent normal group were found to be 75.51% (37/49) and 48.65% (18/37), respectively, with the former showing a significantly higher proportion than the latter (P = 0.01) (**Table 1**).

**Table 1:** Expression of PDIA3 between endometrial cancer and normal tissues by IHC.

Samples	N	PDIA3		χ2	p-value
		Low or None cases	High cases		
Normal	37	19 (51.4%)	18 (48.6%)	6.598	0.01
Cancer	49	12 (24.5%)	37 (75.5%)		

### 3.3 The expression of PDIA3 in endometrial cancer tissues is negatively correlated with clinicopathological features

**Figure 3:**
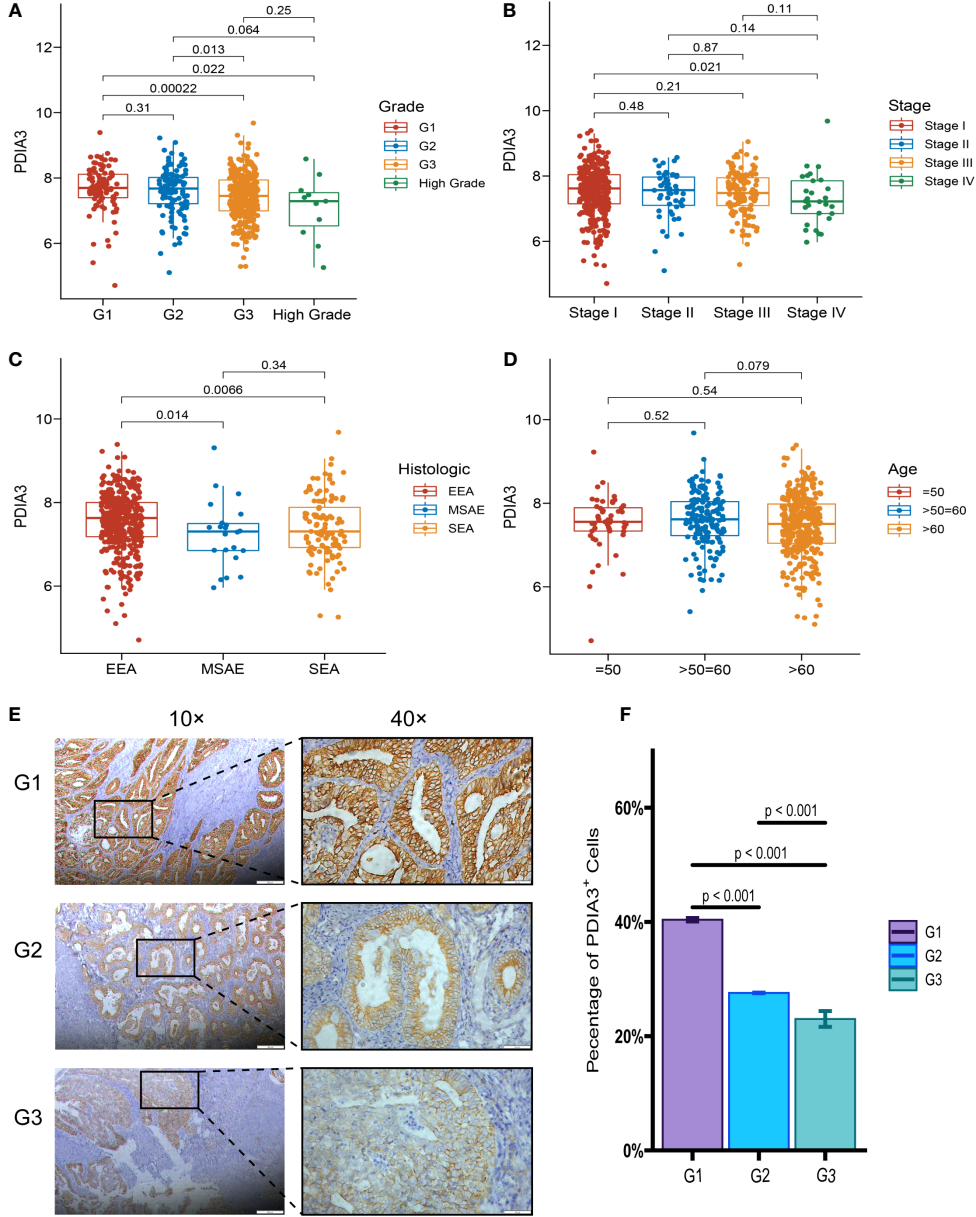
Relationship between PDIA3 expression and clinical features. **(A)** PDIA3 expression in the G1and G2 group was higher than that in the G3 group (P = 0.00022 and P = 0.013, respectively), **(B)** PDIA3 expression was higher in tissues from stage I cancer than in those from stage IV cancer (P = 0.021), **(C)** PDIA3 in endometrioid endometrial adenocarcinoma (EEA) was higher than that of mixed serous and endometrioid (MSAE) (P = 0.014) and serous endometrial adenocarcinoma (SEA) (P = 0.0066), **(D)** Expression of PDIA3 does not differ significantly with age. The expression of PDIA3 in EC IHC associated with the degree of differentiation. **(E)** the expression of PDIA3 in highly differentiated (G1), moderately differentiated (G2), and poorly differentiated (G3) (Magnifications of 10×, with a scale bar of 200 μm and 40×, with a scale bar of 50 μm), **(F)** The percentage of PDIA3 positive expression in highly differentiated, moderately differentiated, and poorly differentiated EC tissue according to IOD/area.

Although the expression of PDIA3 was elevated in endometrial cancer tissues, interestingly, the expression of PDIA3 in EC tissues was inversely correlated with clinicopathological parameters. An analysis was conducted in TCGA datasets to examine the relationship between PDIA3 expression and clinical features. The results indicated that PDIA3 expression showed an inverse correlation with advanced histologic grade. The G1 and G2 groups exhibited higher PDIA3 expression compared to the G3 group (P = 0.00022 and P = 0.013, respectively) (**Figure 3A**). Additionally, PDIA3 expression was higher in EC tissues from stage I than in those from stage IV (P = 0.021) (**Figure 3B**). Among different tissue types, the expression of PDIA3 in endometrioid endometrial adenocarcinoma (EEA) was higher than that in mixed serous and endometrioid (MSAE) (P = 0.014) and serous endometrial adenocarcinoma (SEA) (P = 0.0066) (**Figure 3C**). However, there was no significant difference in PDIA3 expression based on age (**Figure 3D**). The intensity of staining in EC tissue, as observed in the IHC analysis, varied significantly according to the degree of differentiation. Well-differentiated EC tissue (G1) exhibited a strong positive reaction, whereas moderately differentiated EC tissue (G2) displayed an intermediate staining between High-differentiated (G1) and poorly differentiated (G3) samples. Poorly differentiated (G3) EC tissue only showed weak positive reaction (**Figure 3E**). The differences among the three groups were all statistically significant (p< 0.001) (**Figure 3F**).

Based on IHC analysis, the positive expression rate of PDIA3 in EC patients with stages I–II was 83.33% (30/36), which was higher compared to patients with stages III–IV (53.85%, 7/13) (P = 0.034). PDIA3 protein expression demonstrated a strong positive relationship with the degree of differentiation. As the degree of differentiation decreased, the rate of PDIA3 expression gradually decreased. Specifically, the expression rate in the well-differentiated group (G1) was 100% (9/9), slightly higher than the moderately differentiated group (G2) (83.3%, 20/24) (P = 0.024), and much higher than the poorly differentiated group (G3) (50%, 8/16) (P = 0.012). PDIA3 expression was higher in the group without lymph node metastasis compared to the group with lymph node metastasis (χ2 = 5.593, P = 0.018). PDIA3 expression was strongly related to clinical stage and N stage (χ2 = 4.491, P = 0.034), but no significant relationship was observed with patient age, T stage, and distant metastasis (P > 0.05) (**Table 2**). Spearman analysis revealed that high expression of PDIA3 was negatively related to pathologic differentiation (P = 0.002), clinical stage (P = 0.032), N classification (P = 0.034), and lymphatic invasion (P = 0.018) (**Table 3**).

**Table 2:** The relationship between PDIA3 and clinicopathological characteristics (IHC).

Characteristics	N	PDIA3		χ2	p-value
		Low or none cases	High cases		
Age (year)					
<60	30	9 (30.0%)	21 (70.0%)	0.618	0.432
≥60	19	3 (15.8%)	16 (84.2%)		
Pathologic					
G1	9	0 (0%)	9 (100%)		0.26*
G2	24	4 (16.7%)	20 (83.3%)	5.079	0.024**
G3	16	8 (50.0%)	8 (50.0%)		0.012***
Clinical stage					
I	28	4 (14.3%)	24 (85.7%)	4.491	0.034#
II	8	2 (25.0%)	6 (75.0%)		
III	9	4 (44.4%)	5 (55.6%)		
IV	4	2 (50.0%)	2 (50.0%)		
T classification					
T1	29	5 (17.2%)	24 (82.8%)		0.538*
T2	9	2 (22.2%)	7 (77.8%)		0.272**
T3	11	5 (45.5%)	6 (54.5%)	3.386	0.066***
N classification					
N0	36	6 (16.7%)	30 (83.3%)	4.491	0.034
N1	13	6 (46.2%)	7 (53.8%)		
Metastasis					
M0	45	10 (22.2%)	35 (77.8%)	0.399	0.528
M1	4	2 (50.0%)	2 (50.0%)		
Lymphatic invasion					
No	37	6 (16.2%)	31 (83.8%)	5.593	0.018
Yes	12	6 (50.0%)	6 (50.0%)		

* The comparison of G1 and G2 or T1 and T2; **the comparison of G2 and G3 or T2 and T3; *** the comparison of G1 and G3 or T1 and T3; # the comparison of I-II and III-IV.

**Table 3:** Correlation between PDIA3 and clinicopathological characteristics.

Variables	PDIA3 expression level	
	Spearman Correlation	p-value
Age	0.042	0.774
Pathologic differentiation	-0.432	0.002
Clinical stage	-0.307	0.032
T classification	-0.240	0.097
N classification	-0.303	0.034
Metastasis	-0.177	0.224
Lymphatic invasion	-0.338	0.018

The relationships between PDIA3 expression and clinical features on TCGA datasets are presented in **Table 4**. PDIA3 expression exhibited a positive correlation with the degree of differentiation. The expression of PDIA3 in the G1 and G2 groups was higher than that in the G3 group (P = 0.00345). Moreover, PDIA3 expression in endometrioid endometrial adenocarcinoma was higher than in mixed serous and endometrioid and serous endometrial adenocarcinoma (P = 0.0455).

**Table 4:** PDIA3 Expression with clinical features in TCGA database.

Characteristics	Total (n=535)	PDIA3 Expression		P-value
		High (n=197)	Low (n=338)	
Age (year)				
< 65	283 (52.9%)	104 (52.8%)	179 (53.0%)	0.928
≥ 65	250 (46.7%)	92 (46.7%)	158 (46.7%)	
Unknown	2 (0.4%)	1 (0.5%)	1 (0.3%)	
Stage				
I	333 (62.2%)	126 (64.0%)	207 (61.2%)	0.729
II	50 (9.3%)	19 (9.6%)	31 (9.2%)	
III	123 (23.0%)	44 (22.3%)	79 (23.4%)	
IV	29 (5.4%)	8 (4.1%)	21 (6.2%)	
Grade				
G1–G2	216 (40.4%)	96 (48.7%)	120 (35.5%)	0.00354
G3–High Grade	319 (59.6%)	101 (51.3%)	218 (64.5%)	
Histological type				
EEA	400 (74.8%)	158 (80.2%)	242 (71.6%)	0.0455
MSE	22 (4.1%)	4 (2.0%)	18 (5.3%)	
SEA	113 (21.1%)	35 (17.8%)	78 (23.1%)	
Menopause status				
Post	438 (81.9%)	163 (82.7%)	275 (81.4%)	0.647
Pre	69 (12.9%)	26 (13.2%)	43 (12.7%)	
Unknown	28 (5.2%)	8 (4.1%)	20 (5.9%)	
Diabetes				
NO	301 (56.3%)	113 (57.4%)	188 (55.6%)	0.263
YES	111 (20.7%)	34 (17.3%)	77 (22.8%)	
Unknown	123 (23.0%)	50 (25.4%)	73 (21.6%)	
Hypertension				
NO	180 (33.6%)	74 (37.6%)	106 (31.4%)	0.2
YES	263 (49.2%)	87 (44.2%)	176 (52.1%)	
Unknown	92 (17.2%)	36 (18.3%)	56 (16.6%)	
Pregnancies				
0-2	256 (47.9%)	94 (47.7%)	162 (47.9%)	0.00171
3-4+	163 (30.5%)	46 (23.4%)	117 (34.6%)	
Unknown	116 (21.7%)	57 (28.9%)	59 (17.5%)	
Neoadjuvant treatment				
No	534 (99.8%)	196 (99.5%)	338 (100%)	0.784
Yes	1 (0.2%)	1 (0.5%)	0 (0%)	
Radiation therapy				
NO	287 (53.6%)	109 (55.3%)	178 (52.7%)	0.815
YES	224 (41.9%)	80 (40.6%)	144 (42.6%)	
Unknown	24 (4.5%)	8 (4.1%)	16 (4.7%)	

### 3.4 High expression of PDIA3 as an independent protective factor for overall survival

Univariate Cox regression analysis in this study revealed that patient age (≥65 vs. <65), clinical stage (III vs. I; IV vs. I), histological grade (G3-high grade vs. G1-G2), histological type (MSE vs. EEA; SEA vs. EEA), residual tumor (R1-2-X vs. R0), and high expression level of PDIA3 were significantly associated with overall survival (OS) (P < 0.05). Moreover, multivariate Cox regression analysis demonstrated that clinical stage (HR = 2.95, 95% CI [1.7, 5.11], P < 0.001 and HR = 5.82, 95% CI [2.81, 12.05], P < 0.001, respectively) and high expression of PDIA3 remained significantly correlated with OS after adjusting for confounding factors (HR = 0.47, 95% CI [0.27, 0.82], P = 0.008) (**Table 5**). The results of this study demonstrate that the Kaplan-Meier survival curves support the association between high expression of PDIA3 and improved overall survival, indicating a potential protective role of this protein. These findings suggest that PDIA3 protein may serve as an independent protective factor.

**Table 5:** Cox regression for PDIA3 with clinical variables in TCGA database.

Variables	Univariate analysis		Multivariate analysis	
	HR (95% CI)	p	HR (95% CI)	p
Age (≥ 65 vs. < 65)	1.69 (1.11,2.58)	0.015	1.44 (0.91,2.27)	0.116
TNM stage (II vs. I)	2.04 (0.97,4.31)	0.062	1.77 (0.82,3.82)	0.143
TNM stage (III vs. I)	3.62 (2.2,5.94)	<0.001	2.95 (1.7,5.11)	<0.001
TNM stage (IV vs. I)	8.99 (4.93,16.41)	<0.001	5.82 (2.81,12.05)	<0.001
Histologic grade	3.62 (2.08,6.3)	<0.001	1.73 (0.92,3.26)	0.09
(G3–High Grade vs. G1–G2)				
Histological type (MSE vs. EEA)	2.84 (1.21,6.66)	0.016	1.81 (0.74,4.4)	0.193
Histological type (SEA vs. EEA)	2.89 (1.86,4.48)	<0.001	1.28 (0.77,2.13)	0.333
Menopause (Pre vs. Post)	0.79 (0.4,1.58)	0.509	–	–
Diabetes (Yes vs. No)	1.3 (0.79,2.14)	0.296	–	–
Hypertension (Yes vs. No)	1.17 (0.73,1.88)	0.518	–	–
Pregnancy (3-4+ vs. 0-2)	1.26 (0.79,2.02)	0.335	–	–
Radiation therapy (Yes vs. No)	0.67 (0.42,1.05)	0.083	–	–
Residual tumor (R1-2-X vs. R0)	2.64 (1.63,4.27)	<0.001	1.14 (0.65,2.02)	0.643
PDIA3 expression (High vs. Low)	0.36 (0.21,0.61)	<0.001	0.47 (0.27,0.82)	0.008

"-" represents no data available.

**Figure 4:**
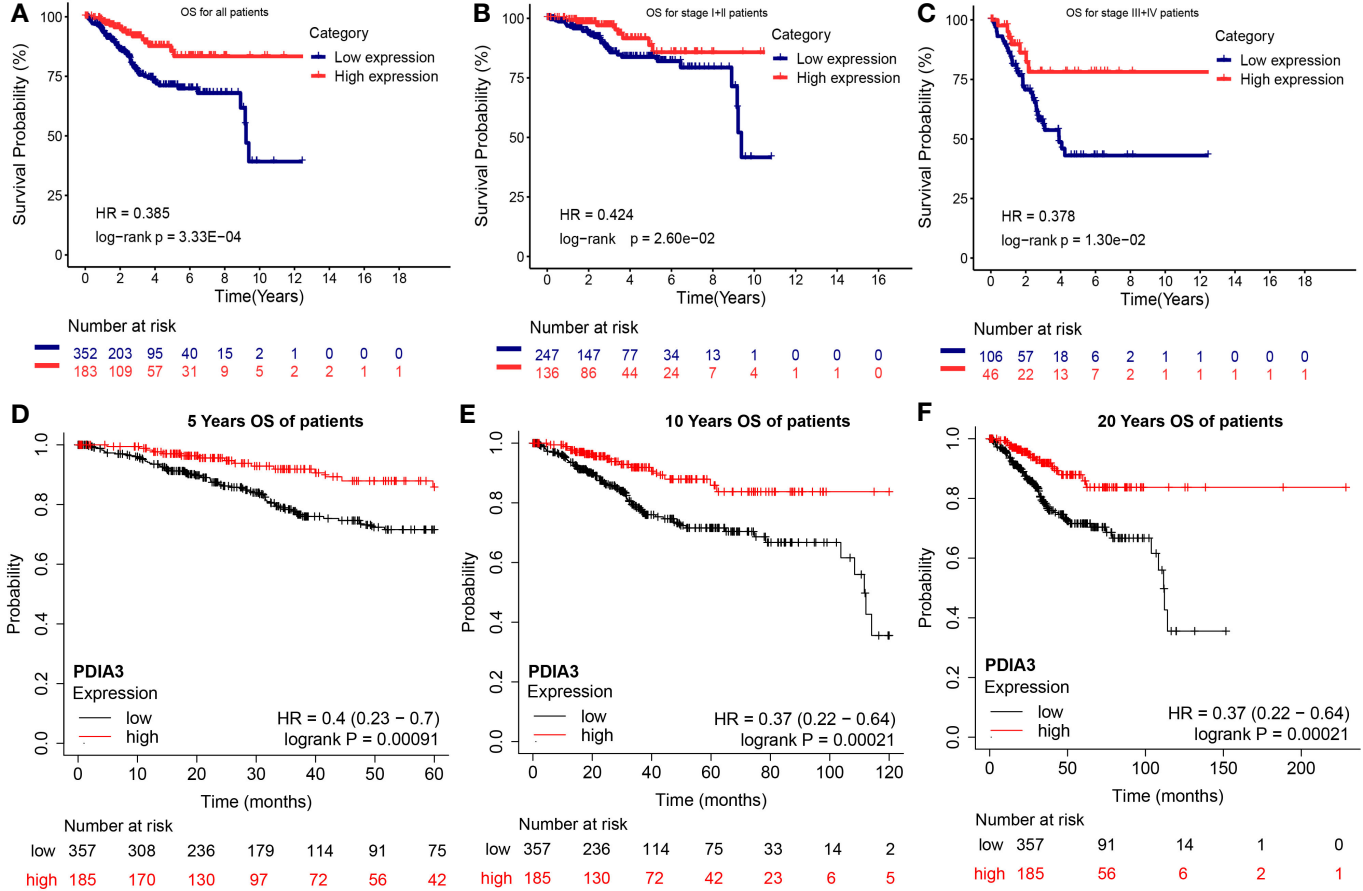
Overall survival (OS) of endometrial cancer with high and low expression PDIA3 gene in TCGA and GEO database. **(A)** OS for all EC patients in TCGA database, **(B)** OS for stage I and II EC in TCGA database, **(C)** OS for stage III and IV EC in TCGA database, **(D)** 5 years OS for EC in GEO database, **(E)** 10 years OS for EC in GEO database, **(F)** 20 years OS for EC in GEO database.

According to the Kaplan–Meier analysis in the TCGA dataset shown in **Figure 4A**, overexpression of PDIA3 was found to be a promising factor for predicting OS in patients with EC. Patients with high expression of PDIA3 had longer OS compared to those with low expression (P = 3.33E-04). Subgroup analysis based on clinical stages indicated that PDIA3 overexpression was associated with better OS in patients across different clinical stages (P = 2.60E-02 and P = 1.30E-02, respectively) (**Figures 4B, C**).

Additionally, Kaplan-Meier analysis, which included the survival information from GEO datasets, further supported the significant predictive behavior of PDIA3 overexpression in the 5-year OS (P = 0.00091), 10-year OS (P = 0.00021), and 20-year OS (P = 0.00021), thus reinforcing the conclusions (**Figures 4D–F**).

### 3.5 Correlation between PDIA3 expression and the level of immune cell infiltration in endometrial cancer

**Figure 5:**
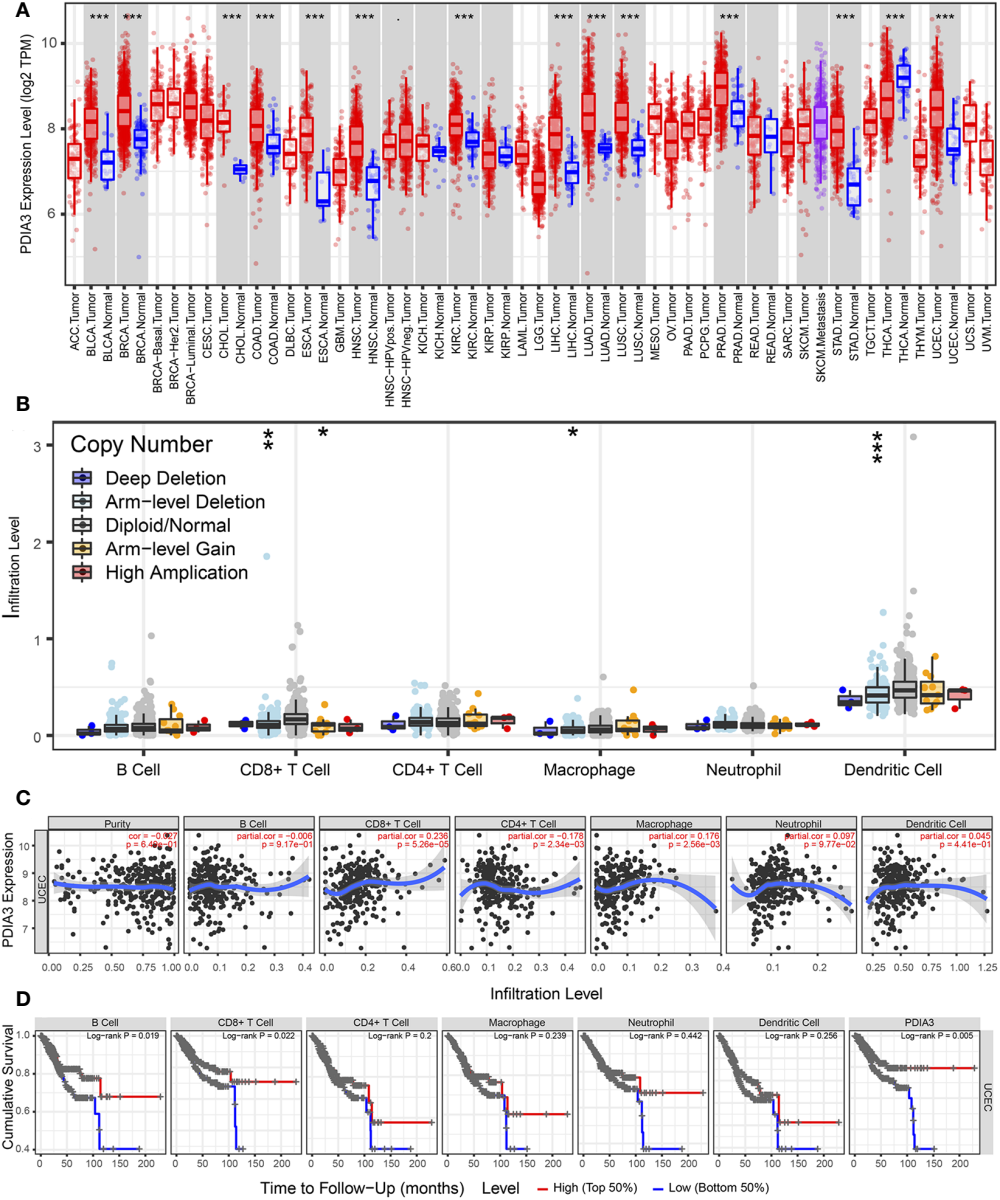
TIMER database analysis PDIA3 gene expression with immune cell infiltration in endometrial cancer. **(A)** PDIA3 gene expression in pan-cancer, **(B)** The distribution of immune cell infiltration of PDIA3 in different SCNA states for pairwise comparison with the normal group and each altered group in EC, P-value Significant Codes: p< 0.001(***), p< 0.01 (**), p< 0.05 (*), p< 0.1 (.), **(C)** the correlation between PDIA3 expression and immune-infiltrating cells in EC, **(D)** The relationship between the expression levels of PDIA3 gene or the levels of immune cell infiltration and patient survival.

According to the results from the TIMER tool (https://cistrome.shinyapps.io/timer/), PDIA3 expression in pan-cancer samples (red box plot) was found to be higher than that in non-tumor samples (blue box plot) in EC specimens (P < 0.001) (**Figure 5A**). As shown in **Figure 5B**, the relationship between PDIA3 and the infiltration levels of six immune cell types in endometrial cancer under different SCNA states is depicted. The comparison of immune cell infiltration levels between each SCNA category and the diploid/normal group was performed using two-sided Wilcoxon rank-sum tests. Furthermore, a clinical correlation analysis was conducted to investigate the relationship between PDIA3 expression in EC and immune-infiltrating cells. The results indicated that PDIA3 was positively correlated with tumor-infiltrating CD8+ T cells (P = 5.26E-05) and macrophages (P = 2.56E-03), while it exhibited a negative correlation with tumor-infiltrating CD4+ T cells (P = 2.34E-03) (**Figure 5C**). In endometrial cancer, the relationship between the expression levels of PDIA3 gene or the levels of immune cell infiltration and patient survival can be visually assessed using Kaplan-Meier survival curves. The survival of patients with EC was found to be correlated with the degree of infiltration of B cells (P = 0.019) and CD8+ T cells (P = 0.022), while the level of PDIA3 expression was negatively correlated with cumulative survival (P = 0.005) (**Figure 5D**).

### 3.6 GSEA: PDIA3-related pathways in endometrial cancer

**Figure 6:**
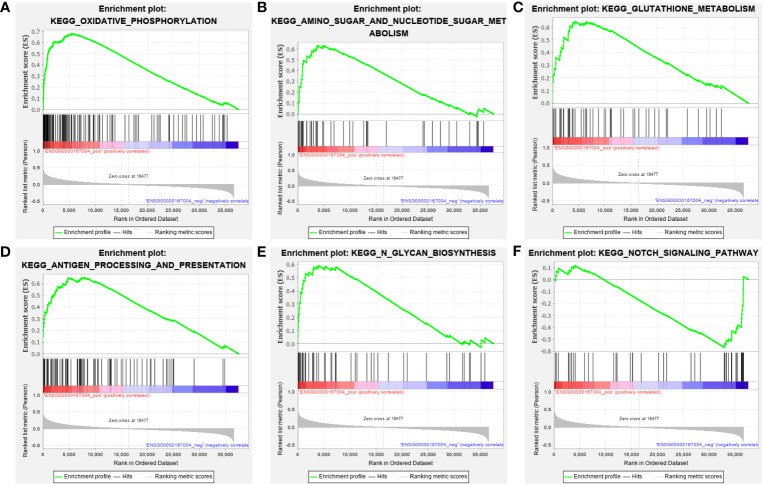
GSEA analysis of the KEGG pathway enrichment for single gene PDIA3. **(A)** Oxidative phosphorylation, **(B)** Amino sugar and nucleotide sugar metabolism pathways, **(C)** Glutathione metabolism, **(D)** Antigen processing and presentation, **(E)** N glycan biosynthesis. **(F)** NOTCH signaling pathway.

To investigate the role of PDIA3 in EC, a Gene Set Enrichment Analysis (GSEA) was performed using samples from EC patients with high and low expression of PDIA3 based on the TCGA dataset. The analysis showed that higher PDIA3 expression in EC samples was positively correlated with several pathways, including Kyoto Encyclopedia of Genes and Genomes (KEGG) oxidative phosphorylation (**Figure 6A**), amino sugar and nucleotide sugar metabolism pathways (**Figure 6B**), glutathione metabolism (**Figure 6C**), antigen processing and presentation (**Figure 6D**), and N glycan biosynthesis (**Figure 6E**). Conversely, PDIA3 expression was negatively associated with the NOTCH signaling pathway (**Figure 6F**).

## 4 Discussion

In this study, we introduced a novel biomarker, PDIA3, for EC and validated its expression using public datasets and primary tumor tissues. We also explored the association between PDIA3 protein expression and the clinical-pathological parameters of EC. Finally, we evaluated the prognostic value of PDIA3 in EC.

Our results revealed that PDIA3 expression in EC tissue was higher than in normal adjacent tissue, suggesting that higher expression of PDIA3 may play a potential role in the development of EC. Additionally, we identified a correlation between PDIA3 expression and clinicopathological parameters. Immunohistochemical analysis showed that patients with PDIA3 overexpression tended to have greater tumor differentiation, while patients with low expression of PDIA3 generally had poor differentiation in pathological tissues. This conclusion aligns with previous studies, such as in cervical cancer where PDIA3 protein expression was found to be negatively correlated with tumor differentiation, and in gastric cancers where PDIA3 protein expression in highly and moderately differentiated tumors was significantly stronger than in poorly differentiated tumors (17, 18).

Furthermore, our research demonstrated that PDIA3 had good prognostic value in EC patients. This finding is consistent with previous studies on esophageal squamous cell carcinoma (19), but contrary to the results reported in hepatocellular carcinoma (20). We attribute this discrepancy to the possibility of tumor heterogeneity, as the same gene may exhibit distinct functions in various diseases.

To further explore the role of PDIA3 in EC, we compared the Gene Set Enrichment Analysis (GSEA) results between high and low PDIA3 expression datasets and analyzed the potential biological functions of PDIA3 through Gene Ontology (GO) terms and Kyoto Encyclopedia of Genes and Genomes (KEGG) pathways. The GSEA findings revealed significant differences in the enrichment of GO terms and KEGG pathways in samples expressing high levels of PDIA3. We selected the signaling pathways with the highest enrichment based on their Normalized Enrichment Score (NES). GSEA data showed that high expression of PDIA3 is primarily involved in oxidative phosphorylation, amino sugar and nucleotide sugar metabolism, glutathione metabolism, antigen processing and presentation, and N-glycan biosynthesis.

Glutathione homeostasis and programmed cell death have been discovered as therapeutic targets due to glutathione depletion, including apoptosis, necrotizing sclerosis, and autophagy in certain diseases (21). Another study also discussed the regulation of mTORC1 activity by certain amino acids (22). These results indicate that crucial pathways involved in the regulation of cellular amino acid metabolism in patients with EC are closely related to the expression of PDIA3.

Research has shown that PDIA3 and calreticulin form a flexible belt of accessory proteins that encircle and stabilize the peptide loading complex (PLC) (23). In the PLC editing module, PDIA3 forms stable disulfide bonds with MHC-I and interacts with tapasin (TPN) to form a rigid core dedicated to peptide editing (24, 25). During the peptide loading process, PDIA3 forms a complex with TPN, connecting MHC-I with the antigen presentation-related transporter protein TAP, ensuring proper peptide loading. The synergistic interaction between MHC-I and PDIA3 ensures the correct loading of appropriate peptides by MHC-I, enabling immune recognition and antigen presentation. Antigen processing and presentation are essential immune functions of dendritic cells and macrophages, necessary for promoting immune responses (26). The surface presentation of MHC class I (MHC-I) molecules to peptides is vital for adaptive responses by CD8 T cells. A defect in the antigen processing and presentation mechanism (APM) acts as an immune escape mechanism, impairing tumor cells’ recognition and killing ability by tumor antigen-specific cytotoxic CD8 T cells (27, 28). Downregulation or loss of PDIA3 can lead to a deficiency in class I MHC expression, allowing tumor cells to evade recognition and destruction by cytotoxic T lymphocytes and other immune cells (29). Considering the role of PDIA3 in the assembly of the MHC class I antigen processing complex, these findings suggest that down-regulation of PDIA3 may contribute to more aggressive tumor behavior. In our study, we identified a positive relationship between overall survival (OS) and high levels of circulating antibodies targeting PDIA3. PDIA3-specific T-cell clones were found to kill tumor cells through a Fas-FasL interaction, indicating their antitumor effector functions (30).

## 5 Conclusion

In summary, our study aimed to investigate the expression of PDIA3 in endometrial cancer and analyze its correlation with clinical parameters and prognosis. Through our analysis, we observed the overexpression of PDIA3 in endometrial cancer tissues, along with its association with tumor grade, prognosis, and other factors. These findings provide preliminary evidence supporting PDIA3 as a potential prognostic biomarker in endometrial cancer. Nevertheless, it is important to acknowledge the limitations of our study. Further mechanistic investigations are required to establish the involvement of PDIA3 in the pathogenesis of endometrial cancer. Additionally, the relationship between PDIA3 and immune regulation remains uncertain and warrants further exploration. Conducting such research will contribute to a comprehensive understanding of the role of PDIA3 in endometrial cancer and its potential as a therapeutic target.

## Data availability statement

The original contributions presented in the study are included in the article/supplementary material. Further inquiries can be directed to the corresponding authors.

## Ethics statement

The studies involving humans were approved by our medical ethics committee of Shanghai Jiao Tong University Affiliated Sixth People’s Hospital South Campus (Ethical code: 2018-KY-17). The studies were conducted in accordance with the local legislation and institutional requirements. The participants provided their written informed consent to participate in this study.

## Author contributions

FY analyzed the bioinformatic data. XW, FY and MG drafted the manuscript. MG have involved in the conception and design of the study. FY, ML and XL performed the experiments. XW, FY and MG drafted the manuscript and edited the manuscript. All authors contributed to the article and approved the submitted version.
